# Clinical and laboratory characteristics of infectious mononucleosis by Epstein-Barr virus in Mexican children

**DOI:** 10.1186/1756-0500-5-361

**Published:** 2012-07-20

**Authors:** Napoleón González Saldaña, Victor Antonio Monroy Colín, Georgina Piña Ruiz, Hugo Juárez Olguín

**Affiliations:** 1 Department of Infectious Diseases, National Institute of Pediatrics, Avenida Insurgentes Sur 3700-C, Mexico City, 04530, Mexico; 2 Laboratory of Pharmacology, National Institute of Pediatrics (Avenida Imán N° 1), 3rd piso Colonia Cuicuilco, CP 04530, Mexico City, Mexico; 3 Department of Pharmacology, Faculty of Medicine, National Autonomous University of Mexico, Mexico City, 04510, Mexico

**Keywords:** Epstein-Barr virus, Mononucleosis, Pediatric infections

## Abstract

**Background:**

Infectious mononucleosis (IM) or Mononucleosis syndrome is caused by an acute infection of Epstein-Barr virus. In Latin American countries, there are little information pertaining to the clinical manifestations and complications of this disease. For this reason, the purpose of this work was to describe the clinical and laboratory characteristics of infection by Epstein-Barr virus in Mexican children with infectious mononucleosis.

**Methods:**

A descriptive study was carried out by reviewing the clinical files of patients less than 18 years old with clinical and serological diagnosis of IM by Epstein-Barr virus from November, 1970 to July, 2011 in a third level pediatric hospital in Mexico City.

**Results:**

One hundred and sixty three cases of IM were found. The most frequent clinical signs were lymphadenopathy (89.5%), fever (79.7%), general body pain (69.3%), pharyngitis (55.2%), hepatomegaly (47.2%). The laboratory findings were lymphocytosis (41.7%), atypic lymphocytes (24.5%), and increased transaminases (30.9%), there were no rupture of the spleen and no deaths among the 163 cases.

**Conclusions:**

Our results revealed that IM appeared in earlier ages compared with that reported in industrialized countries, where adolescents are the most affected group. Also, the order and frequency of the clinical manifestations were different in our country than in industrialized ones.

## Background

Infectious mononucleosis (IM) or Mononucleosis syndrome is caused by an acute infection of Epstein-Barr virus (EBV). It has been reported that about 90% of the cases is caused by EBV [1-3]. The natural infection by EBV occurs only in humans and the result is a life-long infection. In industrialized countries, there is greater possibility of developing mononucleosis if EBV infection occurs in the second decade of life. Seroepidemiological studies have shown that about 91% of all adults worldwide have had first-time infection by EBV. In developing countries, first-time infection by EBV is more frequent in the first decade of life. The incidence of infectious mononucleosis varies in each country in such a way that in U.S.A., 500 cases per 100,000 inhabitants are reported every year with a higher incidence in the age group from 15 to 24 years. Ebell [2], reported a higher incidence of infectious mononucleosis in people from 10 to 19 years old (6 to 8 cases per 1,000 people per year), and a lower incidence in children less than 10 years old (1 case per 1,000 people per year) and a milder clinical manifestations which is frequently underdiagnosed [4-6]. The transmission of EBV is principally through exposition to infected saliva, and for such, it has been denominated “kissing disease”.

The clinical manifestations of infectious mononucleosis are characterized by fever, lymphadenopathy, and pharyngitis and the duration of the infection is about 16 days, while the complications are variable. Hematological complications have been reported in 25% to 50% of the cases. Such complications include hemolytic anemia, thrombocytopenia, aplastic anemia, thrombotic thrombocytopenic purpura, hemolytic uremic syndrome, and disseminated intravascular coagulation [5,6]. Neurologic complications are seen in 1% to 5% of the cases, and include such complications as Guillain-Barre syndrome, facial paralysis, meningoencephalitis, aseptic meningitis, transversal myelitis, peripheral neuritis, cerebellitis and optical neuritis [6]. A potentially fatal complication is splenic rupture, which has been reported in 0.5% to 1% of the cases as well as air way obstruction (1% of the cases) provoked by lymphoid hyperplasia and mucosal edema [6]. In some occasions, EBV serves as a trigger for the development of hemophagocytic lymphohistiocytosis characterized by prolonged fever, lymphadenopathy, hepatosplenomegaly, exanthem, hepatic dysfunction, and cytopenia. It is calculated that this problem is seen in approximately 1 of every 800,000 people per year, and includes all ages. In Latin American countries, there is little information pertaining to the clinical manifestations and complications of this disease. For this and other reasons previously mentioned, the objective of this work was to describe the clinical and laboratory characteristics of infection by Epstein-Barr virus in Mexican children with infectious mononucleosis.

## Results

**Figure 1:**
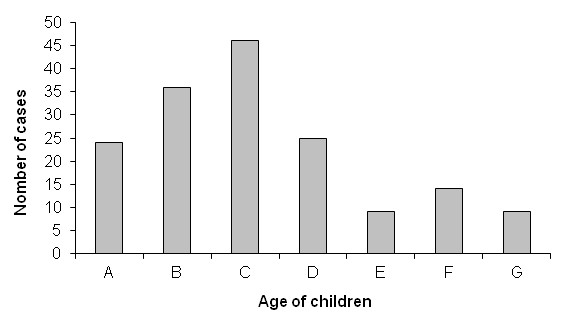
Age of 163 patients included in the study grouped by age-ranges: A:0-2.5 years (y), B:2.5-5 y, C:5-7.5 y, D:7.5-10 y, E:10-12.5 y, F:12.5-15 y, G:15-17.5 y.

Two hundred and eighty three clinical files of patients that were admitted at National Institute of Pediatrics, Mexico City in the aforementioned period with clinical diagnosis of infectious mononucleosis were reviewed. Fulfillment of the inclusion criteria were found in 163 of the patients corroborated through serological studies of Epstein-Barr virus infection. The age range of the patients included in the study was from 2 months to 17 years (Figure 1).

**Figure 2:**
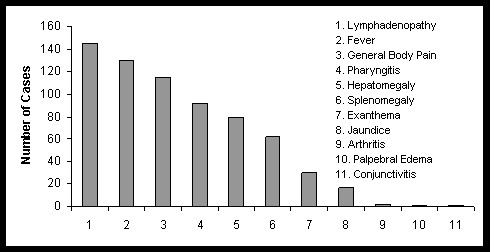
Clinical manifestation of infectious mononucleosis in 163 patients attended at NIP from 1970 to 2011.

The clinical characteristics of the patients were variable and some of these manifestations were present at the time of admission at the Institute. Lymphadenopathy was seen in 146 patients (89.5%); fever in 130 (79.7%); general body pain 113 (69.3%); pharyngitis 90 (55.2%); and hepatomegaly in 77 (47.2%). Other clinical data obtained were: 60 (36.8%) cases with splenomegaly; 27 (16.5%) exanthem; 16 (9.8%) jaundice; 3 (1.84%) arthritis; and 1 (0.61%) conjunctivitis and edema (Figure 2).

From laboratory studies, the data of leucocytes, total lymphocytes, and atypic lymphocytes were obtained on admission. At the same time, the levels of transaminases and bilirubin were registered. From this study, the leukocyte count was between 500 and 41,000/mm^3^ with an average value of 9,424 leukocytes/mm^3^ while total lymphocyte count was between 200 and 31,600/mm^3^ with an average value of 4,248/mm^3^. Atypic lymphocytosis (values of atypic lymphocytes greater than 15%) occurred when leukocyte count were between 6,000 and 41,000/mm^3^. Lymphocytosis defined as lymphocyte count greater or equal to 4,000cells/mm^3^ was found in 68 (41.7%) of the patients (Tables 1 and 2).

**Table 1:** Blood test in 163 patients with infectious mononucleosis by EBV at National Institute of Pediatrics from 1970 to 2011

	**Average**	**Range**
Leukocytes (cells/mm^3^)	9,424	500–41,000
Total Lymphocytes (cells/mm^3^)	4,248	200–31,600

**Table 2:** Lymphocytes and atypical lymphocytosis in 163 patients with Infectious mononucleosis by EBV at National Institute of Pediatrics from 1970 to 2011

	**No. of cases (%) with abnormal values**
Presence of atypical lymphocytes^1^	40 of 163 (24.5%)
Lymphocytosis^2^	68 of 163 (41.7%)

1. Atypical lymphocyte figures greater than 15%.

2. Lymphocytosis defined as lymphocyte count greater or equal to 4,000 cells/mm3.

Test of liver function on admission revealed elevation of transaminase very much above the normal level in 39 (30-9%) of 126 patients. Bilirubin was found in 16 (42.1%) of 38 cases subjected to the test. The determination of bilirubin as well as transaminase was not done in all the cases on admission. It was performed only in patients with clinical data of jaundice and/or hepatitis (Table 3).

**Table 3:** Hepatic function test in patients with infectious mononucleosis by EBV

	**No. of cases (%) with abnormal values**
Transaminases^a^	39 of 126 (30.9%)
Bilirubins^a^	16 of 38 (42.1%)

a. These parameters were not evaluated in all the patients on admission, only in those with clinical data of jaundice and/or hepatitis.

**Figure 3:**
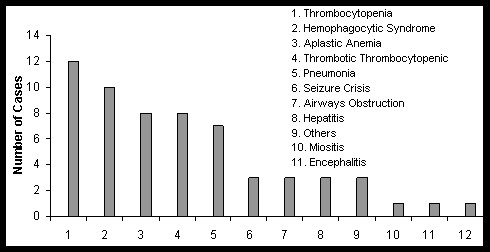
Complications in 62 of 163 patients infectious mononucleosis attended at NIP from 1970 to 2011.

Complications were seen in 52 (38%) of the cases with the principal findings secondary to an infection by EBV being hematologic complications and within which the most frequent were anemia and thrombocytopenia incident in 12 (7.3%) of the patients. Other complications were: Hemophagocytic syndrome in 10 (6.1%); aplastic anemia 8 (4.9%); thrombotic thrombocytopenic purpura in 8 (4.9%); pneumonia 7 (4.3%) cases; air way obstruction 3 (1.8%) cases; hepatitis 3 (1.8%); seizures 3 (1.8%); non-thrombocytopenic purpura in 2 (1.2%); encephalitis 1 (0.6%); myositis 1 (0.6%); lymphoproliferative syndrome 1 (0.6%); and other cases 3 (1.8%) (Figure 3). In this study, lymphadenopathy was found in 89.5%. Table 4 shows an overview of these clinical data and some other reports. No death was registered during the period.

**Table 4:** Comparison of the clinical and laboratory in different studies of patients with infectious mononucleosis secondary to Epstein-Barr virus

**Clinical and laboratory data**	**Authors**				
	**Balfour**^**(9)**^**, 2005 (%)**	**Grotto**^**(10)**^**, 2003 (%)**	**Rea**^**(11)**^**, 2001 (%)**	**Gao**^**(12)**^**, 2011 (%)**	**Present study 2011 (%)**
Lymphadenopathy	95	88.9	57	95	89.5
Fever	30	79	45	92.3	79.7
General body pain	NR	NR	77	NR	69.3
Pharyngitis	100	95	73	83.5	55.2
Hepatomegaly	25	36.7	7	58.1	47.2
Splenomegaly	35	53.3	8	47.4	36.8
Exanthema	17	16.7	15	14.8	16.5
Jaundice	NR	16.7	<10	0	9.8
Myalgia	50	32.4	0	0	0
Palpebral edema	10	NR	0	11.5	0.61
Atypic Lymphocytosis	NA	59.2	85.7	51.9	24.5
Transaminasemia	NA	57.9	31	48.6	30.9
Hyperbilirubinemia	NA	14.9	6	NR	42.1

NR: Not reported. NA: Not analyzed.

## Discussion

Infectious mononucleosis is traditionally characterized by the triad of fever, lymphadenopathy, and pharyngitis. Balfour in his studies described that 100% of the patients with IM present pharyngitis [1]. In this study however, only 55.2% of the cases presented this clinical manifestation while lymphadenopathy was found to be the most frequent clinical sign which was observed in 89.5% of the cases. Moreover, the presence of IM was described in the studies published in industrialized countries as a sickness of the adolescents and young adults [1,7,8], while our study is revealing that the age group mostly affected is constituted by pre-school children with the average age of presentation being 5.2 years old.

It is important to mention that there are no recent studies in Latin American countries that focus on clinical manifestations and complications of infectious mononucleosis in children. However, the present study is demonstrating that the clinical characteristics differ from that reported in industrialized countries. In our study, fever was found in 70.7% of the cases which was different from 34% and 45% reported in the studies of Balfour and Grotto respectively [1,9]. Pharyngitis was seen only in half of the patients (55.2%) in this series while in the study of Balfour [1], it was seen in 100% of the group and only 73% in the study of Rea [10]. In the case of lymphadenopathy, we found that 89.5% of the cases had this manifestation which was similar to that reported by Balfour [1], Grotto [9], and Gao [11], who observed 95%, 88.0%, and 89.5% respectively in their study.

With respect to the findings in physical exploration, hepatomegaly was found in 47.2% while Balfour [1], reported 25%, Grotto [9] 36.7%, and Rea 7% [10]. For splenomegaly, our result of 36.8% was similar to that reported by Balfour with 35% [1], varying greatly with that of Grotto [10], who reported 53.3% and Gao [11], with 47.4% while Rea [10], described only 7%. Our finding of 16.5% cases of exanthem was similar to that described by the authors [1,9-11]. However, the similitude of our result for jaundice with 9.8% rammed with the data of 10% reported by Rea [10].

The presence of atypic lymphocytes above 15% is highly an indicative of IM by EBV. In this study, 24.5% of the cases presented this result which was similar to the reports of other authors [12]. The elevation of transaminase common to IM was seen in 30.9% of our cases, a figure markedly less than that reported in other studies where the figure was up to 80% [13-15]. Hyperbilirubinemia, seen in 42.1% of our patients, was far different from the result of Grotto [9], who found this alteration in 14.9%. It is important to point out that transaminase and bilirubin values were only measured in patients with clinical data of jaundice and/or hepatitis.

Finally, the most frequent complications found in our study were anemia and thrombocytopenia found in 7.3% of the cases. Others were aplastic anemia, thrombotic thrombocytopenic purpura, hemophagocytic syndrome, lymphoproliferative syndrome and hepatitis. Although in the literature, splenic rupture was described as complication (with a frequency between 0.5% and 1%) [1,16,17], in this study and in our country in general, it is not a complication seen in children, probably because the presentation of mononucleosis is in younger children and the risk of suffering from trauma is less in comparison with what occurs in industrialized countries.

Infectious mononucleosis in developing countries is seen in earlier ages in comparison with what occurs in industrialized countries where the incidence is higher in adolescence. Moreover, the clinical signs occur with differential frequency and the complications are distinct with the triad of fever, lymphadenopathy, and pharyngitis being the most frequent as in developed countries.

## Conclusions

Our results revealed that IM appeared in earlier ages compared with that reported in industrialized countries, where adolescents are the most affected group. Also, the order and frequency of the clinical manifestations were different in our country than in industrialized.

## Methods

All cases diagnosed as infectious mononucleosis secondary to Epstein-Barr virus with a compatible clinical manifestation and serological confirmation in children attended at National Institute of Pediatrics (NIP), Mexico City from 6th November, 1970 to 30th July, 2011 were included in the study. The research protocol was approved by the Ethics Committee of NIP, Mexico. The clinical files of all the patients with the aforementioned diagnosis in the period under study were retrospectively reviewed and 163 cases were detected

Age, sex, and clinical characteristics as fever, conjunctivitis, palpebral edema, jaundice, exanthem, splenomegaly, hepatomegaly, pharyngitis, general body pain, and lymphadenopathy as well as data about hemoglobin, leucocytes, lymphocytes, atypic lymphocytes, platelets, alanine aminotransferase, aspartate aminotransferase, and bilirubin were obtained. Also, patients who either had or did not have complications associated to the infection by the virus as well as the mortality related to it, were evaluated. In order to have uniform medical criteria, we considered as a case of mononucleosis all patients with fever, pharyngitis, and/or lymphadenopathy with positive serology of infection by Epstein-Barr virus which was considered as confirmation of the diagnosis when at least one of the following markers: Viral capsid antigen Immunoglobulin M (VCA IgM), Viral capsid antigen Immunoglobulin G (VCA IgG), anti early antigen (anti-EA) is positive. In one case, the diagnosis was confirmed by in situ hybridization in biopsy of lymphatic node. After capturing the data, their statistical descriptions were elaborated and their frequencies and averages were presented with their respective graphical representations.

## Competing interests

This manuscript has no financial competing interest.

## Authors’ contribution

NGS contributed substantially to conception and design. VAM contributed substantially to acquisition of data and analysis. GPR contributed substantially to acquisition and interpretation of data. HJO was involved in drafting the manuscript or revising it critically for important intellectual content. All authors read and approved the final manuscript and have given final approval of the version to be published.
